# Supplementary material for: Utility of PROM Questionnaires: Correlation of Question Burden and Response Rate Among Surgically Treated Patients with Musculoskeletal Diseases
Source: J Clin Med. 2025 Sep 24;14(19):6728. doi: 10.3390/jcm14196728 (PMC12525256; doi:10.3390/jcm14196728)
Supplement: Supplementary file 1 [file jcm-14-06728-s001.zip › jcm-3859892-supplementary/Supplementary S1--additional_statistical results_PROM.pdf]

Supplementary S1: further details of the statistical analyses and materials

Materials

Table S1. Answering patterns (completed PROMs).

Preop	3 kk	1 v	N	%
			261	11.4 %
x			300	13.1 %
	x		74	3.2 %
		x	113	4.9 %
x	x		207	9.0 %
x		x	240	10.5 %
	x	x	192	8.4 %
x	x	x	908	39.6 %

Table S2. Total number of answers. The first number has been calculated using the results with a completed PROM (i.e., numerical result for the main PROM). The second number (in parenthesis) also includes the questionnaires which were started but not sufficiently completed in order to calculate the numerical PROM value.

	N	%
**No answers**	261 (241)	11 % (11 %)
**1 answer**	487 (452)	21 % (20 %)
**2 answers**	639 (643)	28 % (28 %)
**all 3 answers**	908 (959)	40 % (42 %)

Table S3. Electronic and non-electronic answers. The first number has been calculated using the results with a completed PROM (i.e., numerical result for the main PROM). The second number (in parenthesis) also includes the questionnaires which were started but not sufficiently completed in order to calculate the numerical PROM value.

	Electronic	Paper	Did not answer
**PREOP**	1169 (1174)	486 (507)	640 (614)
**3 months**	904 (935)	477 (489)	914 (871)
**1 year**	969 (1015)	484 (495)	842 (785)

Table S4. Electronic and non-electronic answers in percentages. The first number has been calculated using the results with a completed PROM (i.e., numerical result for the main PROM). The second number (in parenthesis) also includes the questionnaires which were started but not sufficiently completed in order to calculate the numerical PROM value.

	Electronic	Paper	Did not answer
**PREOP**	50.9 % (51.2 %)	21.1 % (22.1 %)	27.9 % (26.8 %)
**3 months**	39.4 % (40.7 %)	20.8 % (21.3 %)	39.8 % (38.0 %)
**1 year**	42.2 % (44.2 %)	21.1 % (21.6 %)	36.7 % (34.2 %)

Table S5. Number of answers received after SMS notifications.

	1st SMS	2nd SMS
**PREOP**	909 (913)	197 (198)
**3 months**	661 (679)	236 (249)
**1 year**	718 (747)	239 (256)

Table S6. Number of questions in each register and the answering percentages. Note that the age distributions etc. differ between the registers making the interpretation less trivial. Registers with large differences between pre- and post-op percentages have been highlighted. After adjusting for the multiple tests, there are four registers with significant evidence against a difference between pre- and post-op answering percentages. For knee prothesis the willingness to answer increases slightly, while for spinal and upper limb registers the opposite is true. However, the obvious conclusion here is that, having 75 questions pre-op in the ankle register scares people off from answering the post-op questionnaires. Pre-op this register has one of the higher answering percentages but post-op it has the very lowest percentage of answers. The comparison was carried out using Fisher's exact test. There were N=167 individuals in the ankle register, so this difference does not affect the modeling results significantly and it is best to stick to the current model. The upper limb register is not included in the models since the ASA-classes are not available. However, this register is studied graphically separately. For the two post-op measurements the answering-% were 37% and 35% in the ankle register, so no further drop-off is seen after the effect of the massive pre-op questionnaire.

Register	# Questions (PRE)	PRE %	# Questions (3 mo/1 yr)	POST %	TOTAL %	p-value (PRE & POST differ)	Adj. p-value (Bonferroni, 10 tests)
**Shoulder instab.**	54	60%	37	37%	44%	0.20	1
**Shoulder rot. cuff**	54	73%	37	68%	70%	0.57	1
**Shoulder arthr.**	52	72%	35	73%	73%	>0.99	1
**Hip arthr.**	12	78%	12	85%	83%	0.01	0.10
**Hip**	12	47%	12	38%	41%	0.56	1
**Knee arthr.**	**12**	**78%**	**12**	**86%**	**83%**	**0.001**	**0.01***
**Knee**	42	64%	42	47%	53%	0.03	0.32
**Foot and ankle**	**75**	**72%**	**58**	**36%**	**48%**	**<0.001**	**<0.001*****
**Spine**	**38**	**71%**	**31**	**64%**	**66%**	**0.002**	**0.02***
**Hand**	**45**	**67%**	**33**	**41%**	**50%**	**<0.001**	**<0.001*****
