# Supplementary material for: Utility of PROM Questionnaires: Correlation of Question Burden and Response Rate Among Surgically Treated Patients with Musculoskeletal Diseases
Source: J Clin Med. 2025 Sep 24;14(19):6728. doi: 10.3390/jcm14196728 (PMC12525256; doi:10.3390/jcm14196728)

Supplementary S2: further details of the statistical analyses of the results

Results

The age in the model is the chronological age minus 60 and the number of questions is the actual number of questions minus 48.

Table S7. Results from the model for primary responses.

		Estimate	SE	p-value
**Fixed linear effects**	Constant	0.467	0.104	$8\times 10^{-6***}$
	Female	0.111	0.071	0.12
	ASA = 2	-0.035	0.102	0.73
	ASA = 3	-0.258	0.119	0.03*
	ASA = 4	-1.676	0.325	$3\times 10^{-7***}$
	Number of questions	-0.021	0.002	$<2\times 10^{-16***}$
**Fixed smooth effects**	Age			$<2 \times 10^{-16***}$
**Random effects**	ID	σ = 0.60		
	Register	σ = 0.61		

Smoking was found to be a good predictor of unwillingness to answer a questionnaire. After controlling for smoking, sex was no longer a statistically significant predictor. It should be noted that from those whose smoking status was recorded, 24.6% of males and 14.6% of females smoked. Though ASA is not statistically significant predictor, it should be noted that this model was fitted using only those 50% of data, which had the smoking status included. Since several registers did not record the smoking status, some registers are missing completely. The model did not converge when the number of questions was included as a covariate, and thus it was left out.

Table S8. The results for the explorative model for investigating tobacco smoking as a predictor.

		Estimate	SE	p-value
**Fixed linear effects**	Constant	0.953	0.129	$2\times 10^{-13***}$
	Female	-0.060	0.099	0.55
	ASA = 2	0.001	0.132	0.99
	ASA = 3	0.209	0.164	0.20
	ASA = 4	-0.511	0.471	0.28
	Smoking	-0.395	0.13	0.002**

	Number of questions	0.009	0.004	0.03*
**Fixed smooth effects**	Age			0.002**
**Random effects**	ID<br>Register	σ = 0.43<br>σ = 0.61		

Figure S1. These figures show how the answering percentage correlate with age when also the ASA class is also taken into account.

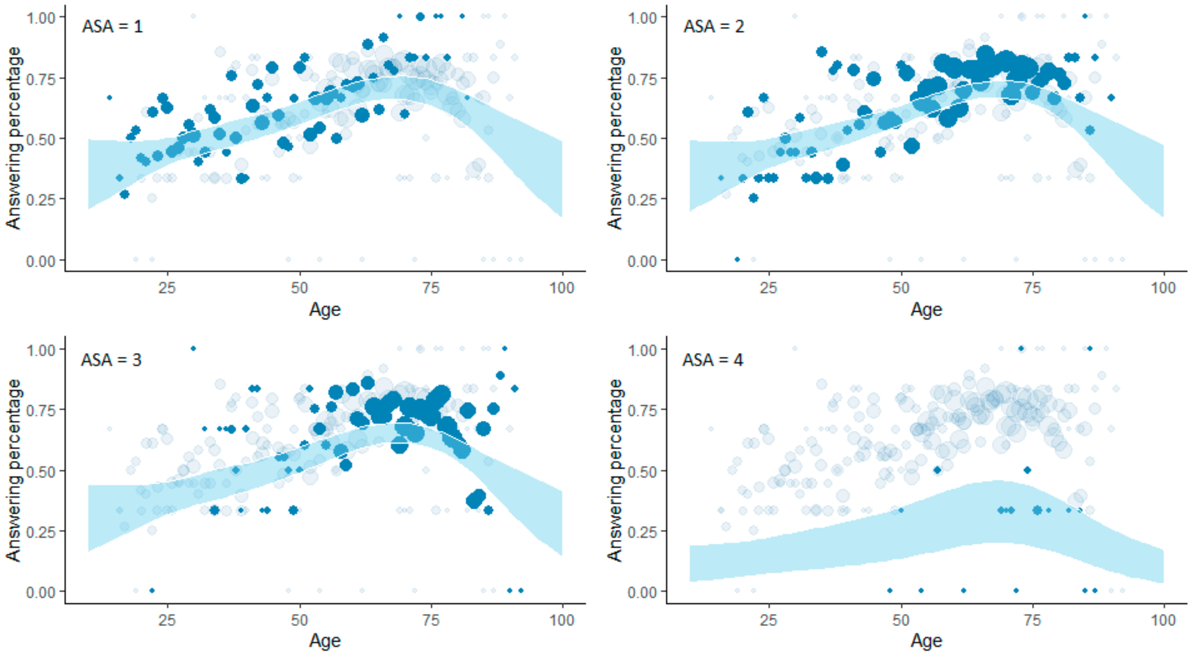

Figure S2. These figures show how the answering percentage correlate with age in the registers 5 and 9.

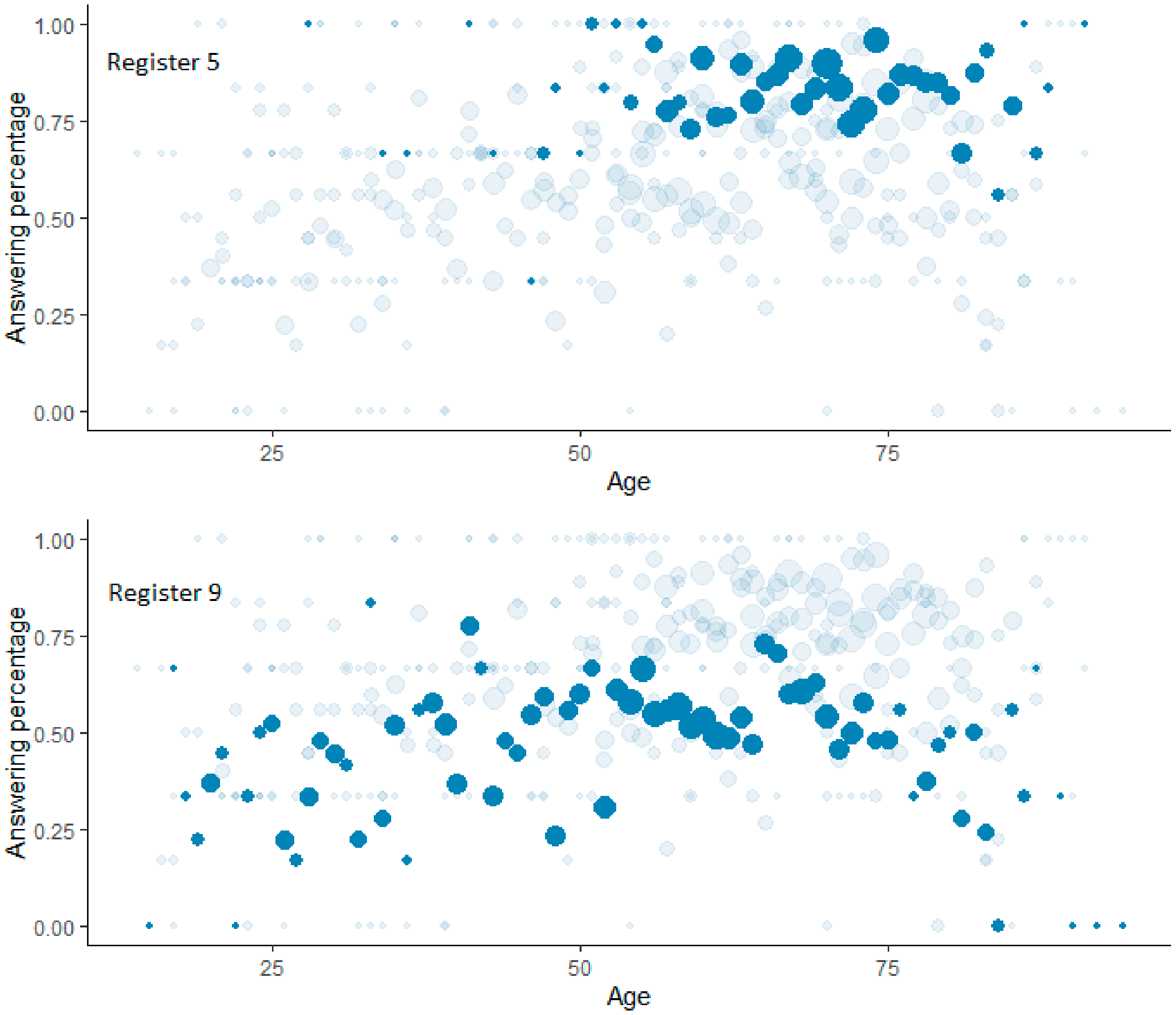

Supplement: Supplementary file 1 [file jcm-14-06728-s001.zip › jcm-3859892-supplementary/Supplementary S2--additional_statistical results_PROM.pdf]
